# Herpes simplex encephalitis as a complication of neurosurgical procedures: report of 3 cases and review of the literature

**DOI:** 10.1186/s12985-016-0540-4

**Published:** 2016-05-23

**Authors:** David A. Jaques, Spyridoula Bagetakou, Arnaud G. L’Huillier, Andrea Bartoli, Maria-Isabel Vargas, Joel Fluss, Laurent Kaiser

**Affiliations:** Division of General Internal Medicine, Geneva University Hospitals, Rue Gabrielle-Perret-Gentil 4, 1205 Geneva, Switzerland; Division of General Pediatrics, Child and Adolescent Department, Geneva University Hospitals, Geneva, Switzerland; Laboratory of Virology, Division of Infectious Diseases and Division of Laboratory Medicine, Geneva University Hospitals, Rue Gabrielle-Perret-Gentil 4, 1205 Geneva, Switzerland; Neurosurgery Division, Department of Clinical Neurosciences, Geneva University Hospitals, Geneva, Switzerland; Neuroradiology Department, Geneva University Hospitals, Geneva, Switzerland; Pediatric Neurology Unit, Pediatric Subspecialties Service, Geneva University Hospitals, Geneva, Switzerland; University of Geneva Medical School, Geneva, Switzerland

**Keywords:** Herpes simplex virus, Meningitis, Encephalitis, Neurosurgery, Complication, Postoperative

## Abstract

**Background:**

Herpes simplex virus (HSV) is the most common identified cause of focal encephalitis worldwide. However, postoperative HSV encephalitis (HSVE) is a rare complication of neurosurgical procedures and a significant clinical challenge

**Method:**

We describe 3 cases of postoperative HSVE and review all published reports. A total of 23 cases were identified.

**Discussion:**

Clinical heterogeneity represents a diagnostic challenge in the postoperative setting. Cerebral magnetic resonance imaging showed typical findings in a minority of patients only, whereas HSV-specific polymerase chain reaction on the cerebrospinal fluid proved to be a valuable test. The postoperative viral pathophysiology remains a subject of debate. The rate of adverse outcome is high and early antiviral treatment seems to be a strong predictor of clinical outcome.

**Conclusion:**

We recommend early empirical treatment for any patient presenting with post-neurosurgical lymphocytic meningo-encephalitis, and prophylactic antiviral treatment for patients with a history of previous HSVE who will undergo a neurosurgical procedure.

## Key points

We report 3 cases of postoperative herpes simplex encephalitis and review the existing literature. The importance of early empiric treatment is highlighted. We recommend prophylactic treatment before neurosurgery for patients with a history of previous herpetic encephalitis.

## Background

Herpes simplex virus (HSV) is the most common identified cause of acute and focal sporadic encephalitis in the Western world with an incidence of 1–3 cases per million inhabitants each year [1]. Without treatment, mortality can be as high as 70 % as opposed to 30 % with adequate treatment and neurological sequelae are frequent, even in treated cases [1]. It has traditionally been recognized that HSV-1 is associated with encephalitis, whereas HSV-2 is a predominant cause of aseptic meningitis. However, a significant overlap exists and more than 15 % of patients with HSV-2 central nervous system (CNS) infection present with encephalitis rather than meningitis [2]. HSV infection is characterized by peripheral nervous system latency and potential reactivation from quiescent episome triggered by various stimuli [3]. It is estimated that approximately two-thirds of HSV encephalitis (HSVE) cases occur because of reactivation, rather than primary infection [4]. HSVE triggered by neurosurgical procedures *per se* is a rare occurrence and deserves special attention. Initial management is challenging because early therapy is critical in a setting where serious intracranial infections are caused almost exclusively by iatrogenic bacterial species. In this article, we present 3 post-neurosurgical HSVE cases diagnosed and managed at our center, review the literature on this subject, and propose diagnostic and management recommendations.

## Case presentations

### Case 1

**Fig. 1:**
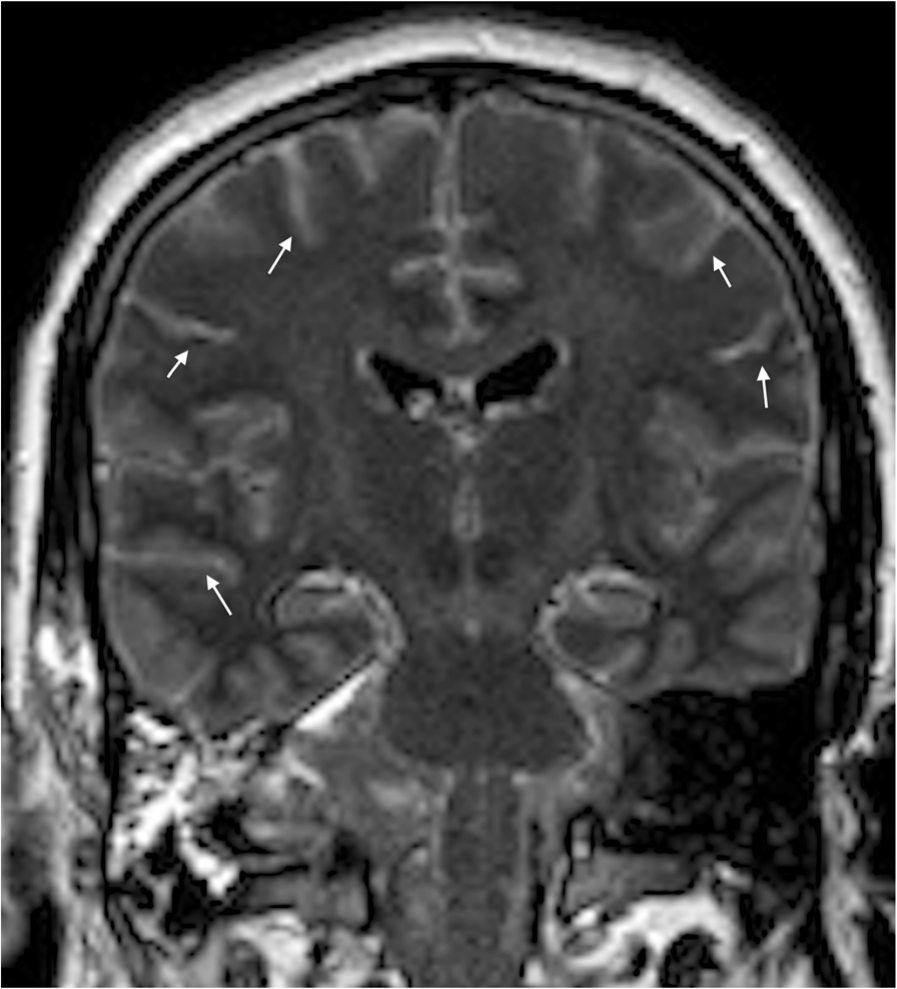
Coronal FLAIR MRI sequence illustrates a diffuse leptomeningeal enhancement (arrows) after surgery of an epidermoid cyst of the right cerebellopontine angle

A 24-year-old man underwent right lateral sub-occipital craniotomy for resection of an epidermoid cyst of the right cerebellopontine angle. Evolution was uneventful and he was discharged at postoperative day (POD) 4 under dexamethasone (4 mg two times per day) for one week. On POD 8, he experienced fever associated with worsening headache. He sought medical attention and was treated with intravenous (IV) ceftriaxone, flucloxacillin and metronidazole (POD 9). The following day, he developed agitated behavior and a decreased level of consciousness. Magnetic resonance imaging (MRI) showed leptomeningeal enhancement in the supra and infratentorial regions without evidence of abscess or empyema (Fig. 1). A lumbar puncture showed lymphocytic meningitis: 1517 M/l leucocytes, 95 % lympho-monocytes, 1 M/l erythrocytes, 0.94 g/l protein, 2.7 mmol/l glucose and 3.2 mmol/l lactate. He was intubated during 24 h and treatment was modified to IV meropenem, vancomycin and acyclovir 10 mg/kg three times daily (POD 11). Cerebrospinal fluid (CSF) cultures remained negative, while the specific polymerase chain reaction (PCR) was positive for HSV-1. Antibiotics were stopped on POD 15 and the patient improved progressively under acyclovir. Subarachnoid hemorrhage with hydrocephaly developed on POD 24. An arteriography showed a ruptured, right anterior inferior cerebellar artery pseudo-aneurysm of traumatic origin attributed to the surgery. Acyclovir treatment was stopped after 21 days. After a 2-month stay, he was discharged with improving right peripheral facial palsy secondary to the hemorrhagic complication and presented no clinical sequelae of the HSV-1 infection.

### Case 2

**Fig. 2:**
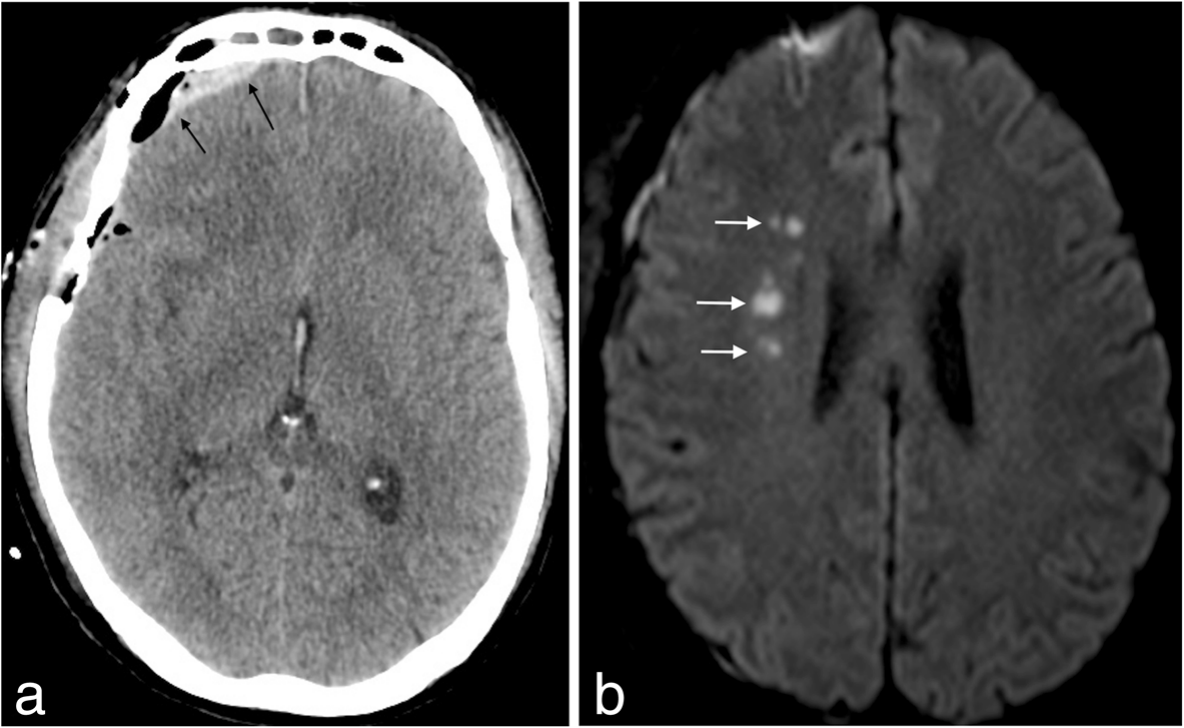
Post-surgical CT visualization of a right frontal heterogeneous collection (2a, left). MRI showed no empyema but ischemic lesions of the right deep frontal white matter (2b, right)

A 53-year-old man underwent an uncomplicated right pterional craniotomy with total resection of a World Health Organization grade I craniopharyngioma. He was discharged on POD 9 under dexamethasone (3 mg three times daily) with subsequent dose tapering. On POD 18, the patient experienced fever and was drowsier than usual. A computed tomography scan showed a right frontal subdural collection; an empyema could not be excluded (Fig. 2a). However, an MRI showed no sign of empyema but instead leptomeningeal enhancement and lesions compatible with acute ischemic changes in the right corona radiata and centrum semiovale (Fig. 2b). CSF analysis showed lymphocytic meningitis with 188 M/l leucocytes, 92 % lympho-monocytes, 26 M/l erythrocytes, 1.25 g/l protein, 1.6 mmol/l glucose, and 4.2 mmol/l lactate. No bacteria were detected on direct examination of the fluid. He was started on IV meropenem, vancomycin, and acyclovir 15 mg/kg three times daily (POD 19). The next day, revision surgery was performed with cranial flap removal. Operative status was normal and showed no sign of empyema. Surgical samples showed no bacteria on direct examination and cultures remained negative. Broad-range PCR for bacteria on surgical samples was negative and vancomycin and meropenem were stopped on POD 22 and 27, respectively. HSV-2 DNA PCR came back positive on the CSF. IV acyclovir was continued for a total duration of 21 days and he improved rapidly. Neurological status at discharge was comparable to baseline.

### Case 3

**Fig. 3:**
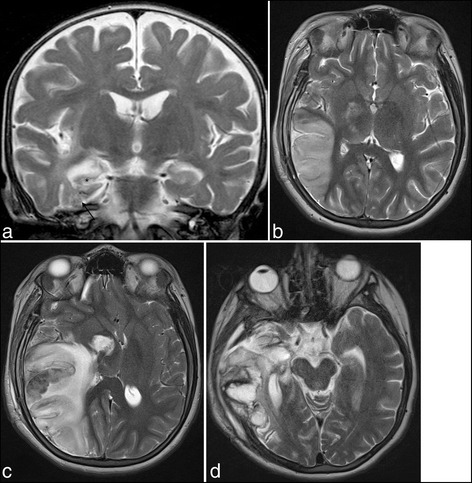
Sequels of HSVE at the level of the temporal lobe and right hippocampus (3a, upper left, arrows and asterisk). Post-surgical MRI shows areas of suspected encephalitis with high signal on T2 in the right frontal, parietal and temporal lobes at 3 weeks (3b, upper right), associated with hemorrhagic transformation and mass effect one month later (3c, lower left). Follow-up MRI at 45 days showed large sequelae of the temporal lobe (3d, lower right)

A young girl suffered from HSV-1 encephalitis at the age of 11 months. By that time, she had predominant involvement of her right temporal lobe in the form of a multiple area of focal encephalomalacia (Fig. 3a) and exhibited clinically minimal left-sided weakness. Over the years, her epilepsy had worsened and became progressively intractable. At 12 years of age, epileptic surgery was considered. She underwent a right temporal lobectomy and amygdalohippocampectomy and was discharged at POD 7 without steroids. At POD 11, she presented to the emergency department for headache associated with fever. A worsening level of consciousness at POD 14 prompted a cerebral MRI that demonstrated abnormal signal intensity with vasogenic edema distant from the resected area and compatible with an inflammatory process (Fig. 3b). CSF analysis revealed mild pleocytosis with 91 M/l leucocytes, 96 % lympho-monocytes, 837 M/l erythrocytes, 2.05 g/l protein, 1.9 mmol/l glucose and 2.7 mmol/l lactate. Empiric IV ceftriaxone and vancomycin were first initiated for presumed superficial wound infection and contiguous bacterial cerebritis. Considering possible HSV relapse, acyclovir 20 mg/kg three times daily was started 24 h later. Positive HSV-1 PCR on the CSF confirmed the diagnosis. Four days after the initiation of acyclovir (POD 18) and after a transient improvement, she complained of severe headache and became confused. An urgent MRI revealed a malignant edema with a significant mass effect (Fig. 3c) prompting a rapid decompressive right frontoparietotemporal craniectomy (Fig. 3d). Thereafter, evolution was fortunately favorable. Her neurological status returned to baseline except for a worsening of the preexisting left hemiplegia. Acyclovir was stopped after 21 days, without recurrence of encephalitis.

## Methods

### Literature review

We identified cases in PubMed without any exclusion criterion, using the key words “herpes”, “herpetic”, “meningitis” or “encephalitis” and one of the following terms: “postoperative”, “surgery”, “neurosurgery” or “craniotomy”. All identified articles and the cited references were searched to identify any other cases.

## Results

A total of 23 cases of HSVE were identified (Table 1). There seems to be no age predisposition as postoperative HSVE can manifest in the pediatric population [5–10], and in adults [6, 11–26]. A minority of patients only had a previous history of HSVE [5, 7, 8, 10, 15, 16, 25]. Most adult patients had uncomplicated neurosurgery for various types of brain tumor. Other surgical indications were: Chiari malformation type 1 [13], refractory epilepsy secondary to previous or presumed HSVE [15, 16, 25, 26] and refractory trigeminal neuralgia [21]. Regarding pediatric cases, neurosurgery was performed for refractory epilepsy secondary to a previous HSVE in 4 patients [5, 7, 8, 10], and CNS tumor for 2 [6, 9]. Time-to-symptom onset was highly variable and varied from only a few hours [21] to 3 weeks [18] post-surgery. Postoperative steroids were prescribed in all cases (when documented).

**Table 1:** Reported Cases of HSVE after Neurosurgery

Author, Year	Age	Previous HSVE History	Diagnosis	Procedure	Time to Symptoms	Etiology	Steroids	Treatment (Time to treatment)	Outcome
Fearnside [6], 1972	41	No	Pituitary adenoma	Craniotomy	POD 4	HSV	Yes	Idoxuridine IV (POD 8)	Death
Fearnside [6], 1972	11	No	Pituitary adenoma	Craniotomy	POD 8	HSV	Yes	None	Death
Perry [19], 1998	64	No	Cranio-pharyngioma	Craniotomy	POD 2	HSV-2	Yes	Acyclovir IV (POD >14)	Cognitive and visual sequelae
Spuler [24], 1999	78	No	Parasagittal meningioma	Craniotomy	POD 10	HSV-1	Yes	None	Death
Bourgeois [5], 1999	8	Yes	Refractory epilepsy	Craniotomy	POD 6	HSV-1	NA	Acyclovir IV (timing not shown)	Complete recovery
Molloy [18], 2000	22	No	Medullo-bastoma	Craniotomy	POD >21	HSV	Yes	None	Death
Lellouch [10], 2000	8	Yes	Refractory epilepsy	Craniotomy	POD 6	HSV-1	NA	Aciclovir (timing not shown)	Speech impairment
Sheleg [23], 2001	28	No	Gliobalstoma multiforme	Craniotomy	POD 2	HSV-1	Yes	None	Death
Aldea [11], 2003	28	Possible	Anaplasic oligo-dendroglioma	Craniotomy	POD 7	HSV-1	Yes	Acyclovir IV (POD 9)	Complete recovery
Filipo [12], 2005	33	No	Acoustic neuroma	Mastoidectomy	POD 2	HSV-1	Yes	Acyclovir IV (POD 11)	Complete recovery
Ploner [20], 2005	47	No	Meningioma	Craniotomy	POD 10	HSV	Yes	Acyclovir IV (POD 13)	Apathic state
Kwon [9], 2008	13	No	Cranio-pharyngioma	Craniotomy	POD 15	HSV	Yes	Acyclovir IV (POD 22)	Speech and motor impairment
Jalloh [14], 2009	44	No	Acoustic neuroma	Mastoidectomy	POD 1	HSV-1	NA	Acyclovir IV (POD 11)	Complete recovery
Ihekwaba [13], 2009	35	No	Type 1 Chiari malformation	Sub-occipital craniectomy	POD 14	HSV-2	Yes	Acyclovir IV (POD >21)	Complete recovery
Gong [7], 2010	2	Yes	Refractory epilepsy	Craniotomy	POD 5	HSV-1	Yes	Acyclovir IV (POD 5)	Complete recovery
Lund [16], 2011	19	Yes	Frontal lobe epilepsy	Craniotomy	POD 10	HSV	NA	Acyclovir (POD 20)	Death
Raper [22], 2011	65	No	Ependymoma	Laminectomy	POD 5	HSV-1	Yes	Acyclovir IV (POD 8)	Complete recovery
Mallory [17], 2012	49	No	Acoustic neuroma	Craniotomy	POD 10	HSV-1	Yes	Valacyclovir PO (POD 10)	Complete recovery
Uda [25], 2013	20	Yes	Medial temporal lobe epilepsy	Craniotomy	POD 11	HSV	NA	Acyclovir IV (POD 11)	Complete recovery
Kim [8], 2013	11	Yes	Refractory epilepsy	Craniotomy	POD 5	HSV-1	NA	Acyclovir IV (POD 10)	Complete recovery
Prim [21], 2013	78	No	Trigeminal neuralgia	Rhizothomy	POD 1	HSV-1	NA	Acyclovir IV (POD 17)	Neuro-psychiatric sequelae
Vik-Mo [26], 2014	25	Possible	Medial temporal lobe epilepsy	Craniotomy	POD 3	HSV-2	NA	Acyclovir IV (POD 18)	Speech impairment
Presti [15], 2015	17	Yes	Refractory epilepsy	Craniotomy	POD 6	No virus found	Yes	Acyclovir IV (POD 11)	Motor and behavioral sequelae
Jaques, case 1 2015	24	No	Epidermoid cyst	Craniotomy	POD 8	HSV-1	Yes	Acyclovir (POD 11)	Complete recovery
Jaques, case 2, 2015	53	No	Cranio-pharyngioma	Craniotomy	POD 18	HSV-2	Yes	Acyclovir IV (POD 19)	Complete recovery
Jaques, case 3, 2015	12	Yes	Refractory epilepsy	Craniotomy	POD 11	HSV-1	No	Acyclovir IV(POD 14)	Mild left hemiparesy

## Discussion

### Background

The vast majority of CNS infections complicating neurosurgery are of bacterial origin and postoperative HSVE remains an exceptional situation, yet not one to miss. Reported cases show great clinical heterogeneity regarding age, type of surgery, and symptom delay (Tables 1 & 2). This non-specific clinical picture can make early diagnosis difficult and delay appropriate treatment. This is all the more important since postoperative HSVE severity seems comparable to sporadic cases with unfavorable outcome in more than half of patients (Table 3).

**Table 2:** Clinical Characteristics of HSVE after Neurosurgery

Age (mean; range)	32.1; 2-78
Previous HSVE history	8/26 (30.1 %)
Time-to-symptoms in days (mean; range)	7.7; 1-21
HSV-2 etiology	4/26 (15.4 %)
Time to treatment in days (mean; range)	5.8; 0-16
Death or sequelae	14/26 (53.8 %)

**Table 3:** Clinical Outcome of HSVE after Neurosurgery

		Death or sequelae
Overall		14/26 (53.8 %)
	Children	4/7 (57.1 %)
	Adults	10/19 (52.6 %)
No treatment^a^		5/5 (100.0 %)
Treatment		9/21 (42.9 %)
	Initiatied ≤ 2 days^b^	0/5 (0.0 %)
	Initiated ≥ 3 days^b^	8/14 (57.1 %)

^a^idoxuridine considered as “no treatment”

^b^2 reports excluded as timing of treatment is not indicated [5, 10]

### Clinical aspects

Several aspects of the above-reported cases are worth considering. First, the latency observed between the surgery and the first symptoms was longer in patient 2 (POD 18) than most of the previous cases described and only 1 other case showed a longer latency (POD >21) [18]. This illustrates that there is no definite time frame and the physician must consider HSVE as a potential diagnosis, even several weeks after initial surgery. Second, only 3 other cases of postoperative HSV-2 CNS infection have been described [13, 19, 26] (Table 2), 2 of which [19, 26] presented with encephalitis. This emphasizes that benign aseptic meningitis is not the sole manifestation of HSV-2 CNS infection. This is supported by a recent study showing that more than 15 % of HSV-2 sporadic CNS infection can present with encephalitis, rather than meningitis [2]. Of note, similar to the case reports of Vik-Mo et al. [26] and Perry et al. [19], patient 2 had no prior history of labial or genital herpetic infection. However, such a history is found only in a minority of patients and thus should not lower the physician’s pre-test probability of HSVE [27].

### Imaging

CNS imaging is an essential step in the diagnostic work-up and characteristic findings of medial temporal lobe and insular involvement on neuroimaging studies contribute significantly to the diagnosis of HSVE. MRI is regarded as the modality of choice in this setting showing early findings with a high sensitivity. It should be emphasized however, that atypical or even normal findings may be found in HSVE, especially in the early stage of the disease [28]. In one series of clinically and biologically diagnosed HSVE, temporal lobe involvement was present in 60 % of cases only and up to 25 % of patients had normal studies (CT or MRI) [29]. The situation seems to be similar in the postoperative setting where patients have been reported to have characteristic [20], atypical [22], or even normal [7, 14] presentations. Cerebral MRI can be difficult to interpret after neurosurgery. Postoperative changes and blood derivatives can mimic infections in diffusion sequences and enhancement can be observed due to breakdown of the hematoencephalic barrier. MRI proved to be of significant help in the diagnostic procedure of our patient 3 where it showed findings compatible with HSVE. Brain imaging showed only aspecific findings in the other two patients, similar to most cases reported previously. Thus, normal postoperative brain imaging should not prevent the clinician from considering HSVE in situations where there is a reasonable clinical suspicion.

###  Cerebrospinal fluid

CSF analysis in sporadic HSVE typically shows a lymphocytic pleocytosis with normal glucose and a normal or mildly-raised protein level, but normal findings can be observed early in the course of the disease [30]. A small number of erythrocytes are also frequently found in the CSF, potentially reflecting the hemorrhagic nature of the disease [31]. Compared to sporadic cases, CSF findings in the postoperative setting seem not to differ significantly. Lymphocytic pleocytosis was the dominant pattern observed in previous reported cases, but normal findings have also been described [20]. Our first two cases had only marginal amount erythrocytes in the CSF, whereas our patient 3 showed a much higher erythrocyte count, potentially prefiguring the dramatic forthcoming hemorrhaging transformation. CSF interpretation in the postoperative setting can be further complicated by the fact that surgery itself can induce aseptic inflammatory meningitis. Whereas brain biopsy was previously regarded as the gold standard to make a definite diagnosis of HSVE, HSV-1/2 PCR on the CSF is the most efficient test with a sensitivity and specificity of >95 % and >99 %, respectively [32]. Most previously reported cases were diagnosed by CSF PCR and no further diagnostic procedure was needed when this examination was performed. CSF analysis showing a typical lymphocytic meningitis pattern in all of our 3 cases argued strongly against a classical bacterial infection. Similar to other reported cases, HSV PCR positivity on the CSF led us to the diagnosis of HSVE. We consider this method reliable as we used a fully certified in-house non-commercial PCR assay. In patient 1, follow-up CSF analyses were ordered in the setting of a neurosurgical complication. After 14 days of IV acyclovir treatment, HSV-1 PCR turned negative. However, it was found to be weakly positive on 2 later occasions (22 and 30 days after treatment onset, respectively). These findings were not correlated with HSVE clinical deterioration and prompted no specific management. Persistence of PCR positivity despite appropriate antiviral treatment has been described and while most patients show CSF PCR negativity after 1 or 2 weeks of treatment, some can retain PCR positivity for as long as 35 days [33]. In this setting, PCR positivity could represent remnant viral DNA. This finding has not been consistently correlated with poor outcome [34].

### Physiopathology

The postoperative viral pathophysiology of HSVE remains a subject of debate. HSVE can be caused by a primary infection by the time of surgery or, more commonly, by a relapse of previous herpetic infection. A clinical relapse seems to harbor two distinct entities [35]: The first is thought to be a post-infectious immune-inflammatory disorder without associated viral replication, while the second involves resumption of active viral replication. This reactivation phenomenon can itself be explained by two distinct mechanisms [1]: a) reactivation of the virus in the trigeminal ganglion with subsequent retrograde axonal transport into the CNS; and b) *in situ* reactivation in the CNS tissue itself where the virus can be found in a latent form. This last hypothesis could also account for cases without clinically overt past HSVE as viral DNA can be detected in the brain of adults without any neurological disease [36]. In most previous postoperative cases however, the exact pathological mechanism is unknown.

Relapse phenomenon is documented in four adults ([15, 16, 25], patient 3) and four pediatric ([5, 7, 8, 10]) cases (Table 2). Among these, only one showed HSV positivity on brain tissue (autopsy) [16]. In the seven other cases ([5, 7, 8, 10, 15, 25], patient 3), direct evidence of cerebral viral presence could not be found or was unavailable. As no evidence of *in situ* latency could be found, reactivation from an extra-cerebral site probably accounts for some of these cases. Peripheral reactivation is also the likely mechanism in one report based on HSV serological status before and after the clinical event [11]. The same mechanism is likely in our patient 2 where HSV-2 DNA PCR was positive on the CSF and standard serological studies showed a pattern consistent with past infection. Serum HSV DNA PCR, intrathecal HSV-2 specific IgG synthesis (Tibbling-Link index), and HSV-1/2 immunostaining, as well as PCR on brain biopsy, were all negative. Globally, these results favor reactivation of the virus in a sensory ganglion with subsequent retrograde axonal transport into the brain as no evidence of primary infection or *in situ* latency could be found. In our patient 3, who presented with HSVE relapse, HSV DNA PCR was negative on the operative specimen, but positive in CSF at the time of diagnosis suggesting that peripheral reactivation started after the surgery. However, the coexistence of an immune-inflammatory process is likely and might explain the unusual diffuse inflammatory change on MRI and the subsequent malignant edema that developed under antiviral therapy. Finally, a pure immune-inflammatory phenomenon without any viral replication is likely in Lo Presti’s patient as HSV DNA was not found in CSF or brain tissue [15]. This is supported by the atypical extensive gray and white matter findings on MRI. An inability to detect viral presence in brain tissue or CSF is not uncommon in HSVE sporadic relapse and led several to speculate an immune-mediated mechanism [37] whereas autoantibodies directed against NMDA receptors have been detected in some patients during the course of an initial episode of HSVE [38]. Armangue et al. recently studied eight teenagers and adults with HSVE relapse and showed that 5 had CSF antibodies against NMDA receptors and three against unknown neuronal cell surface proteins whereas CSF PCR for HSV was negative in all patients. Accurate characterization of the underlying pathophysiological mechanism could prove important as prompt immunotherapy resulted in substantial neurological improvement in these patients [39].

Classical stimuli triggering HSV reactivation include fever, local trauma, physical or emotional stress, exposure to ultraviolet light, hormonal imbalance, and immunosuppression [3]. In agreement with previous reports, we postulate that surgical stress and trauma, as well as corticosteroid use, may facilitate HSV reactivation in the postoperative period. Minimal stimulation to the trigeminal sensory root seems to be sufficient to reactivate latent HSV in humans [40], while dexamethasone has been shown to induce HSV-1 reactivation *in vitro* in a dose-dependent manner [41]. Patients 1 and 2, but not patient 3, received high-dose dexamethasone after surgery. All previous described cases received steroids, except for eight cases where steroid use was not documented [5, 8, 10, 14, 16, 21, 25, 26].

Finally, host factors might play an important role in HSVE pathophysiology and it has been hypothesized that late relapses could be related to specific immunological defects resulting in a particular susceptibility to HSV cerebral infection [42]. Mutations in the interferon pathway, and more specifically in UNC93B1 and TLR3 genes, seem to confer selective susceptibility to HSV infections [43]. Our patient 3 has been tested for these specific mutations but results came back negative.

### Diagnosis

A diagnosis of encephalitis can be accurately defined using the International Encephalitis Consortium case definition [44]. Based on these criteria, our patient 3 meets criteria for confirmed encephalitis with altered level of consciousness, fever, abnormal CSF and imaging as well as documented HSV-1 in CSF. Patients 1 and 2 on the other hand would only qualify for possible encephalitis as imaging showed no specific sign of encephalitis. We still think that encephalitis is more likely than meningitis based on suggestive clinical findings: Patient 1 presented predominantly with agitated behavior and decreased level of consciousness requiring intubation. Moreover, despite rapid improvement under antiviral treatment, opposing behavior and psychomotor slowing persisted for a few days. In patient 2, delirium and temporal disorientation out of proportion with the septic state pointed towards encephalitis rather than meningitis. Of note, electroencephalographic data were unfortunately unavailable for these two patients.

Two elements can explain the atypical imaging in patients 1 and 2. First, the MRI was realized on the same day the encephalitic symptoms began possibly accounting for an early false negative result [28, 29]. Second, a theoretical possibility exists that these two patients suffered from an immune-inflammatory predominant form of relapse. As stated elsewhere [5], findings favoring this hypothesis are the behavioral dominant clinical picture without focal deficit and the atypical neuroimaging results.

### Treatment and outcome

Early treatment is of prime importance in HSVE and represents a major clinical challenge for two reasons. First, the pathological process in the brain is usually well-advanced when patients come to clinical attention and, second, initial signs and symptoms are usually non-specific [30]. Based on the 26 postoperative reported cases, appropriate antiviral treatment seems to be a strong predictor of clinical outcome since death or neurological sequelae were observed in 100 % of untreated patients as opposed to 43 % in the treatment group (Table 3). In addition, complete recovery was universally observed when specific treatment was initiated 2 or fewer days after symptom onset. By contrast, death or neurological sequelae were reported in 57 % of cases when the treatment was administered 3 or more days after symptom onset. This finding is in agreement with the fact that treatment delay was independently associated with poor clinical outcome [45]. In patients 1 and 2, acyclovir treatment was started less than 3 days after symptom onset, thus allowing complete recovery. In patient 3, antiviral treatment was initiated at day 4 after symptom onset, possibly accounting for the mild neurological sequelae. Our three patients have been treated with 21 days of IV acyclovir: 10 mg/kg two times daily, 15 mg/kg two times daily, and 20 mg/kg two times daily (patients 1, 2 and 3, respectively). Given the rarity of postoperative HSVE, no established guidelines exist regarding treatment. For HSV-1 sporadic cases, the Infectious Diseases Society of America 2008 guidelines recommend 10 mg/kg two times daily for 14–21 days in the adult population (20 mg/kg in neonates). Some experts advocate a slightly higher dosage of 15 mg/kg two times daily. Of note, although presenting with encephalitis, patient 2 suffered from HSV-2 infection. As reported recently [27], there is no standard approach to the treatment of sporadic HSV-2 CNS infection. For HSV-2 meningitis, expert opinion usually recommends IV acyclovir 10 mg/kg two times daily for 10–14 days with a possible *per os* switch, but some argue that no treatment is needed for a first and uncomplicated episode. No guidelines exist regarding HSV-2 encephalitis treatment. Since HSV-2 encephalitis can induce neurological sequelae in a significant percentage of cases [2], we recommend to treat these patients as if they had HSV-1 encephalitis.

## Conclusions

Although the vast majority of infectious complications of neurosurgical procedures are of bacterial origin, postoperative HSVE is an established entity. Although HSV-1 is usually the causative agent, HSV-2 can also cause an encephalitic pattern. Given the severity of the disease and the prognostic implication of treatment delay, prompt initiation of IV acyclovir is of prime importance. Thus, we recommend empirical acyclovir treatment in the appropriate clinical setting whenever CSF analysis is consistent with viral meningo-encephalitis. CSF viral-specific PCR together with MRI brain imaging are diagnostic modalities of choice. In agreement with other experts [5, 11, 15, 16], we recommend prophylactic antiviral treatment for patients with an HSVE previous history undergoing neurosurgery. Considering that postoperative HSVE is rare, potentially overlooked and always severe, randomized controlled studies are improbable and clinicians should adapt their clinical practice based on these data.
